# Supplementary material for: The effect of human amnion epithelial cells on lung development and inflammation in preterm lambs exposed to antenatal inflammation
Source: PLoS One. 2021 Jun 25;16(6):e0253456. doi: 10.1371/journal.pone.0253456 (PMC8232434; doi:10.1371/journal.pone.0253456)
Supplement: S1 Fig — Horizontal lines show group mean. (DOCX) [file pone.0253456.s001.docx]

S1 Fig: The number of CD45+ and CD163+ cells and epithelial sloughing scores in the lungs of Sal/Sal, LPS/Sal and LPS/hAEC preterm lambs on day 7 of life. Horizontal lines show group mean.
