# Supplementary material for: The effect of human amnion epithelial cells on lung development and inflammation in preterm lambs exposed to antenatal inflammation
Source: PLoS One. 2021 Jun 25;16(6):e0253456. doi: 10.1371/journal.pone.0253456 (PMC8232434; doi:10.1371/journal.pone.0253456)
Supplement: S2 Fig — (A) The proportion of cells positive for Annexin V increased with time, irrespective of culture temperature (n = 9, performed in triplicate). (B) hAECs positive for 7AAD did not change with culture temperature or time (n = 9, performed in triplicate). Open circles are representative of hAECs cultured at 33°C, grey circles are hAECs cultured at 37°C and black circles are hAECs cultured at 39°C. (DOCX) [file pone.0253456.s002.docx]

S2 Fig. hAECs apoptotic activity is influenced by time, not culture temperatures of 33 °C, 37 °C or 39 °C. (A) The proportion of cells positive for Annexin V increased with time, irrespective of culture temperature (n=9, performed in triplicate). (B) hAECs positive for 7AAD did not change with culture temperature or time (n=9, performed in triplicate). Open circles are representative of hAECs cultured at 33 ºC, grey circles are hAECs cultured at 37 ºC and black circles are hAECs cultured at 39 ºC.
