# Supplementary material for: The effect of human amnion epithelial cells on lung development and inflammation in preterm lambs exposed to antenatal inflammation
Source: PLoS One. 2021 Jun 25;16(6):e0253456. doi: 10.1371/journal.pone.0253456 (PMC8232434; doi:10.1371/journal.pone.0253456)
Supplement: S3 Fig — (A) Percentage change in the wound area recovered by hAECs exposed to culture temperatures 33°C, 37°C and 39°C. (B) Representative images of cells cultured at 33°C, 37°C and 39°C, at 0 and 72 hours (n = 9, performed in triplicate). Open circles are representative of hAECs cultured at 33°C, grey circles are hAECs cultured at 37°C and black circles are hAECs cultured at 39°C. (DOCX) [file pone.0253456.s003.docx]

S3 Fig: hAECs wound healing capacity is the same in 33 °C, 37 °C or 39 °C cultures. (A) Percentage change in the wound area recovered by hAECs exposed to culture temperatures 33 °C, 37 °C and 39 °C. (B) Representative images of cells cultured at 33 °C, 37 °C and 39 °C, at 0 and 72 hours (n=9, performed in triplicate). Open circles are representative of hAECs cultured at 33 ºC, grey circles are hAECs cultured at 37 ºC and black circles are hAECs cultured at 39 ºC.
