# Supplementary material for: The effect of human amnion epithelial cells on lung development and inflammation in preterm lambs exposed to antenatal inflammation
Source: PLoS One. 2021 Jun 25;16(6):e0253456. doi: 10.1371/journal.pone.0253456 (PMC8232434; doi:10.1371/journal.pone.0253456)
Supplement: S4 Fig — M: Molecular sized strands. Lane 20 is positive control. Lane 21 is a negative control. (DOCX) [file pone.0253456.s004.docx]

**
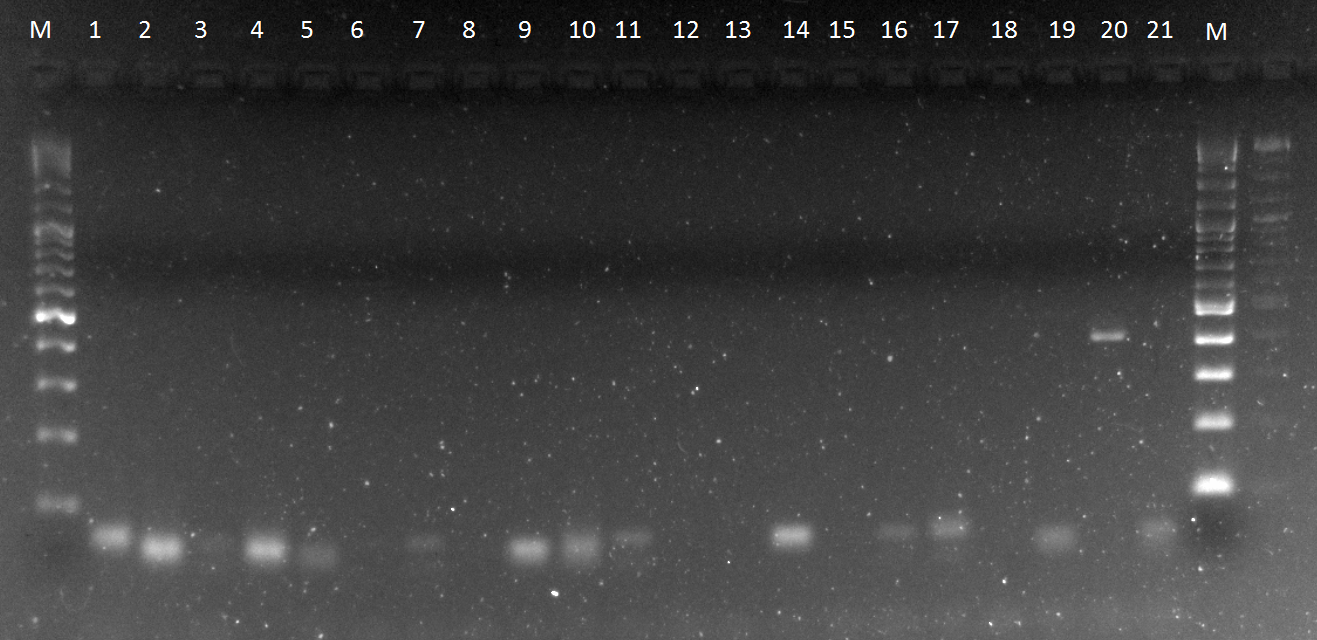
**

S4 Fig: Electrophoretic analysis of PCR Ureaplasma products from 19 hAEC donors. M: molecular sized strands. Lane 20 is positive control. Lane 21 is a negative control.
