# Supplementary material for: The effect of human amnion epithelial cells on lung development and inflammation in preterm lambs exposed to antenatal inflammation
Source: PLoS One. 2021 Jun 25;16(6):e0253456. doi: 10.1371/journal.pone.0253456 (PMC8232434; doi:10.1371/journal.pone.0253456)
Supplement: S1 Table — (DOCX) [file pone.0253456.s005.docx]

| **S1 Table.** Probe selection for TaqMan® Assays | |
| --- | --- |
| **Gene** | **Assay ID/Reference** |
| r18S | Oa4906333_g1 |
| IL-1α | Oa04658682_m1 |
| IL-1β | Oa04656322_m1 |
| IL-4 | Oa04927178_s1 |
| IL-6 | Oa04656315_m1 |
| IL-8 | CUSTOM |
| IL-10 | Oa03212724_m1 |
| TGF-β1 | Oa04259484_m1 |
| TNF | Oa04656867_g1 |
| Surfactant protein A1 | Oa04657758_m1 |
| Surfactant protein B | Oa04750908_g1 |
| Surfactant protein C | Oa04656841_m1 |
| Surfactant protein D | CUSTOM |
| VEGF-A | Oa04653812_m1 |
| VEGFR1 | Oa04694159_m1 |
| Angiopoietin-1 | Oa04757067_m1 |
| EGR1 | Oa03237885_m1 |
| CYR61 | Oa04673852_g1 |
| CTGF | Oa04659069_g1 |
| PECAM | Oa04677168_m1 |
| MMP9 | Oa03215996_g1 |
| MMP12 | Oa04744924_g1 |
| CCL2 | Oa04677078_m1 |
| SAA | Oa04924154_s1 |
| Hepcidin | Oa04656982_m1 |
| Ureaplasma spp | Teng et al., Vol. 32, 1996, JCM, |
| IL = interleukin; TGF = transforming growth factor; VEGF = vascular endothelial growth factor; EGR = early growth response; CYR = cysteine-rich; CTGF = connective tissue growth factor; PECAM = platelet endothelial cell adhesion molecule; MMP = matrix metallopeptidase; CCL = chemokine ligand; SAA = serum amyloid A. | |
