# Supplementary material for: The effect of human amnion epithelial cells on lung development and inflammation in preterm lambs exposed to antenatal inflammation
Source: PLoS One. 2021 Jun 25;16(6):e0253456. doi: 10.1371/journal.pone.0253456 (PMC8232434; doi:10.1371/journal.pone.0253456)
Supplement: S2 Table — (DOCX) [file pone.0253456.s006.docx]

****S2 Table. Fold change mRNA expression of genes in the liver of preterm lambs
