# Supplementary material for: The effect of human amnion epithelial cells on lung development and inflammation in preterm lambs exposed to antenatal inflammation
Source: PLoS One. 2021 Jun 25;16(6):e0253456. doi: 10.1371/journal.pone.0253456 (PMC8232434; doi:10.1371/journal.pone.0253456)
Supplement: S1 File — Detailed methodology outlining animal procedures and the general care of preterm lambs. (DOCX) [file pone.0253456.s007.docx]

**S1 File. Supplementary Methods**

**Human AEC isolation**

All procedures were approved by Monash University Human Ethics Committee (ref #: MUHREC-CF13/2144-2013001109). Placentae were obtained from women undergoing elective term caesarean section. All women provided written informed consent. Exclusion criteria included preterm birth, intrauterine growth restriction, clinical chorioamnionitis or pre-existing maternal disease, including diabetes. The isolation of hAECs from placentas was described previously.^1^ Briefly, the amnion was stripped from adjacent choriodecidua and rinsed in Hanks Balanced Salt Solution (HBSS; Invitrogen, San Diego, CA). Human AECs were isolated by two, 1-hour digests in 0.05 % Trypsin (Invitrogen, San Diego, CA), and collected by centrifugation. Live-cell counts and viability were determined by trypan blue exclusion (>85 % viability was required) at isolation (before cryopreservation) and prior to administration to preterm lambs (immediately after thawing). Human AECs from at least 2 donors were thawed, counted and resuspended in sterile saline (30x10^6^ hAECs in 20 mL) for delivery to preterm lambs at 1 mL/minute.

Flow cytometry

hAECs were seeded at 5 x 10^5^ cells/well on a 6-well plate and placed into separate incubators set at temperatures of 33 °C, 37 °C or 39 °C. Cells were cultured in standard DMEM/F12 media (Gibco, Life Technologies) supplemented with 10% FBS and 1 % antibiotics (Penicillin-Streptomycin, Gibco, Life Technologies), in 5 % CO_2_ in room air. Cultured hAECs (5 x 10^5^ cells) at 33 °C, 37 °C and 39 °C were then harvested at 24, 48 and 72 hours and stained with Annexin V and 7AAD (PE Annexin V Apoptosis Kit I, BD Biosciences, USA) for 15 minutes at room temperature (RT), to assess apoptotic activity. Unstained hAECs were used for controls. Cells positive for Annexin V were considered to be in early apoptosis. Cells positive for 7AAD were considered to be in late apoptosis. Cells were washed with FACS buffer (1 % FBS in PBS) and were centrifuged (Heraeus Megafuge, Thermo Fisher Scientific) at 400 rcf for 5 minutes at 4 °C. Data were acquired with BD FACS-Canto II flow cytometer. Conditioned hAEC medium was collected at 72 hours and stored at -80 °C for future use in phagocytosis assays.

Wound-healing assay

The wound-healing properties of hAECs were assessed with a scratch assay. The hAECs were seeded in DMEM + 10 % FBS + 1 % antibiotics at 5 x 10^5^ cells/well in a 6-well plate and incubated at 37 °C until confluent (7-12 days). A cross was scratched in the middle of the well with a 1,000µl pipette tip at 100 % confluency and hAECs were incubated at temperatures 33 °C, 37 °C or 39 °C for 3 days. Images were taken at the corner of the cross at 0 and 72 hours, using a phase contrast microscope (Axiovert 25, Ziess, Germany) so that the exact position could be replicated. Recovery of the scratch area was analysed using ImageJ.^2^

Phagocytosis assay

The day before phagocytosis assays, immortalised mouse macrophages (iMACs; generously provided by A/Prof Ashley Mansell, Hudson Institute of Medical Research) were plated in DMEM + 10 % FBS + 1 % antibiotics, at a density of 5 x 10^5^ cells/well in a 96-well plate. The next day, media were removed from wells and replaced with 100 µl of FITC-marked fluorescent beads (2 x 10^4^ beads/µl; diameter: 1 µm; Sigma-Aldrich, Fluka, USA) in DMEM + 10 % FCS and 100µl of hAEC-conditioned media (from cultures incubated at 33 °C, 37 °C or 39 °C for 72 hours). As negative controls, iMACs were cultured in 200 µl standard DMEM media, with and without 7AAD. As positive controls, iMACs were cultured in 100 µl standard DMEM media and 100 µl fluorescent beads in DMEM + 10 % FCS, with and without 7AAD.

All treated iMACs were incubated for 3 hours at 37 °C to allow phagocytosis of beads. After incubation, media was removed and iMACs were washed with PBS. Trypsin 0.05 % (100 µl) was used to detach iMACs from the plate; they were resuspended in PBS and centrifuged at 1400 rpm for 10 minutes at RT, and then resuspended in FACS buffer for analysis of positive FITC staining by flow cytometry. 7AAD was added to the relevant controls immediately before flow cytometry data acquisition was performed and analysed using a BD FACS-Canto II flow cytometer. Uptake was expressed as a percentage of fluorescent cells, corresponding to the percentage of iMACs with phagocytosed beads.

**Animals**

The University of Western Australia Animal Ethics Committee approved all animal experimentation (RA 3/100/1454).

Antenatal interventions

Pregnant ewes received intramuscular (IM) medroxyprogesterone (150 mg; Pfizer, Australia) 7 days prior to planned induction of labour, to avoid subsequent betamethasone-induced preterm labour. Ewes received two IM injections of betamethasone (5.7 mg/dose; Celestone, Merck Sharp & Dohme Pty Ltd, Australia) at 48 h and 24 h prior to planned Caesarean section delivery. Pregnant ewes randomly received ultrasound guided intra-amniotic (IA) LPS (4 mg; 2 mg/mL; *Escherichia coli* 055:B5; Sigma-Aldrich, NSW, Australia; n=10) or IA saline (n=10), at 126 days’ gestational age (GA; term ~150d). This dose of LPS results in a well characterised fetal inflammatory response that peaks 48 h after injection.^3^

Preterm delivery

Pregnant ewes at 128 d GA were premedicated with IM buprenorphine (0.01 mg/kg, 300 mcg/mL, Indivor, Pty Ltd, Australia) 1 h prior to IV induction of anaesthesia (15 mg/kg sodium thiopental; Troy Laboratories, Australia). Inhalational anaesthesia (2-3 % Isoflurane; Bomac Animal Health, Australia) was maintained via an endotracheal tube using a ventilator. The fetal head and neck were exposed through maternal laparotomy and hysterotomy. The right carotid artery and right jugular vein of the fetus were catheterised for blood gas sampling and drug administration, respectively. The fetus was intubated and excess lung liquid was passively drained before administration of surfactant (3 mL, 80 mg/mL, poractant alfa, Chiesi Farmaceutici S.pA., Italy) via the endotracheal tube. The umbilical cord was clamped and cut. The lamb was weighed, dried and placed in ventral recumbency, suspended in a sling, on a neonatal baby warmer. Cord blood was collected for blood gas measurement, full blood counts and collections of plasma. The lamb received a sustained inflation (30 cmH_2_O for 30 s) before initiation of ventilation. Body temperature was maintained between 38 ºC and 39 ºC. The ewe was humanely killed immediately after the lamb was delivered (150 mg/kg pentobarbitone; Valobarb, Jurox, Australia).

Postnatal interventions

Human AECs were randomly administered to preterm lambs exposed to IA LPS (LPS/hAEC; 30x10^6^ cells IV; n=7), beginning immediately after delivery. Remaining lambs (delivered after either IA saline or LPS) received postnatal saline (control).

Respiratory support

Ventilated lambs were managed in accordance with best clinical practice in 2017. Over the 7-day experimental period, lambs received graded de-escalation of respiratory support aimed at early weaning from mechanical ventilation (Babylog VN500 Ventilator, Dräger Medical, Lübeck, Germany) onto non-invasive forms of respiratory support. The general pattern involved: mechanical ventilation, then endotracheal bubble continuous positive airway pressure (B-CPAP) by 3-4 hours after birth, then extubation onto nasally delivered heated humidified high flow (HHF; Fisher Paykel Healthcre, Auckland, NZ) or CPAP via nasal cannulae and eventual unassisted breathing of room air. We commenced ventilation with volume guarantee (5-7 mL/kg), a fraction of inspired oxygen (FiO_2_) of 0.3, peak inspiratory pressure (PIP) of 30 cmH_2_O, positive end expiratory pressure (PEEP) of 9 cmH_2_O and ventilator rate of 50 breaths per minute (breaths/min). Ventilatory settings; PEEP, FiO_2_, inspiratory time and rate, ventilation rate and mean airway pressure (MAP) were adjusted with each type of respiratory support (where possible) to target oxygen saturation (SpO_2_) of 90-95 % at the lowest achievable FiO_2_. Amplitude during non-invasive nCPAP and nHHF was adjusted to maintain PaCO_2_ of 45-55 mmHg. FiO_2_ and MAP were adjusted to maintain SpO_2_ of 90-95 %.

Lambs were reintubated if they experienced more than one of the following: 1) ventilatory failure (PaCO_2_ >80 mmHg on 2 consecutive arterial gases >30 min apart, unresponsive to altered nasal amplitude and frequency settings); 2) severe metabolic acidosis; 3) >4 apneas requiring resuscitation within an hour, or 4) persistent SpO_2_ <80 % despite FiO_2_ >0.8 and increased mean airway pressure to a maximum 12 cmH_2_O.

Pulmonary gas exchange was assessed using PaO_2_ to FiO_2_ (pF) ratio and oxygenation index (OI = [(MAP x FiO_2_ x 100)] / [(PaO_2_ x 1.36)].

Figure 1: General postnatal observations in preclinical intensive care research unit for preterm lambs.

General postnatal management

Lambs received daily IV antibiotics twice daily (piperacillin/tazobactam 100 mg/kg, 100 mg/mL, Sandoz Pty Ltd, Australia and gentamicin 6 mg/kg, 100 mg/mL Troy Laboratories, Australia). Caffeine (WAMF, Perth, WA, Australia) was administered IV as a loading dose (20 mg/kg) then daily as required (5mg/kg over ~10 minutes), in accordance with routine neonatal ICU protocol.

Arterial blood samples from the carotid arterial catheter were obtained every 30 minutes for the first hour of life, every hour for the next 4 hours of life, and 4-6 hours thereafter. If the lamb was considered to be stable and on minimal respiratory support, blood gas samples were taken 6-12 hourly. Arterial blood gas samples were used to aid clinical decisions regarding ventilation, fluid management and general lamb well being.

General observations were taken every hour for the duration of experiments Figure 1 shows an observation sheet outlining the parameters recorded each hour.

Lambs received increasing aliquots of enteral feeds (colostrum from ewe) commencing at 2 hours after birth, given at 2-hour intervals for 24 hours, followed by 50:50 feeds (ewe colostrum:lamb formula; Maxcare®, Qld, Australia). With time, lambs were encouraged to suckle and feed from a syringe or bottle. Feeds increased by 1 mL every 6 hours for the first 72 hours, then by 1 mL every 4 hours thereafter.

Cerebral tissue oximetry (Fore-sight absolute tissue oximeter, CAS Medical Systems Inc., CT, USA) was recorded hourly. Urine and stool outputs were recorded over the 7 days of postnatal life.

All observations were recorded by individuals blinded to the treatment interventions of preterm lambs. Hourly observations were entered and managed using REDCap electronic data capture tools (hosted at The University of Western Australia, Perth, Australia). REDCap (Research Electronic Data Capture) is a secure, web-based application designed to support data capture for research studies, providing: 1) an intuitive interface for validated data entry; 2) audit trails for tracking data manipulation and export procedures; 3) automated export procedures for seamless data downloads to common statistical packages; and 4) procedures for importing data from external sources (REDCap, version 7.6.10 © Vanderbily University, Nashville, Tennessee).

Post mortem measurements and tissue collection

On day 7 of life, lambs were killed with an overdose of sodium pentobarbitone (150 mg/kg; Jurox Pty Ltd, Australia). The left lung was inflation-fixed at 30 cmH_2_O with 10 % formaldehyde^4^ for morphometric and histological analyses. Static compliance was calculated as the volume of fixative infused at 30 cmH_2_O divided by the birth-weight. Three random sections of fixed left lung and 5 regions of interest (ROI) per section were used for all histological and immunohistochemical analyses. A total of 15 ROIs at x40 magnification (unless otherwise stated) were analysed per animal. Small subpleural segments of the right lower lobe of the lung and the midline of the liver were frozen in liquid nitrogen for molecular analysis. All analyses were carried out by P.C. Papagianis who was blinded to treatment interventions.

Morphometric analyses

Three random sections of fixed left lung were chosen, and 5 regions of interest (ROI) per section were used for histological analyses. A total of 15 ROIs were analysed per animal.

*Hart’s elastin stain*

Hart’s resorcin-fuschin elastin stain was used to identify tissue and airspace fractions, elastin fibres present on secondary septa of alveoli, and total elastin content. Paraffin-embedded sections of lung tissue were de-waxed with xylene (2 x 5 minutes), rehydrated with absolute ethanol (3 x 3 minutes), rinsed in tap water (3 x 30 seconds), immersed in 0.25 % potassium permanganate (5 minutes) and rinsed in distilled water (2 x 30 seconds). Tissue was briefly submerged in 5 % oxalic acid to dissolve potassium permanganate and then rinsed in tap water. Sections were stained with Hart’s resorcin-fuschin stain for elastin, incubated for 6 hours, rinsed in tap water (3 x 30 seconds) and counterstained with 0.25 % tartrazine in saturated picric acid (3 minutes). Sections were dehydrated with xylene (2 x 5 minutes) and mounted in disyrene plasticiser xylene (DPX; Brittish Drug House Chemicals, United Kingdom). Hart’s elastin stain shows elastic fibres in black and tissue in yellow.

*Quantification of Hart’s elastin stain*

Five random ROIs per section of fixed lung were captured at X20 magnification and used to quantify tissue and airspace fraction and septal crest density. A linear point counting grid was overlaid onto each ROI. Points placed 10 pixels apart, with a total 676 points per ROI. Points that overlaid tissue, air and septal crests were identified using a partly automated ImageJ plugin (Copyright © 2015, Keith Schulze, Monash Micro Imaging, Monash University; NIH Image). The plugin automatically detects points that fall on tissue in green and points that fall on airspace in grey, based on yellow and white colour, respectively. Points falling on septal crests are manually counted.

The points falling on lung tissue or airspace were divided by the total points (676) in the ROI, so as to express the areal fraction of tissue or airspace in that ROI. Septal crest areal fraction was determined by number of points falling on septal crests divided by total points falling on tissue. The mean tissue, airspace and septal crest areal fractions were calculated for each animal.

Total elastin deposition was calculated with a separate automated ImageJ plugin (Copyright © 2015, Keith Schulze, Monash Micro Imaging, Monash University) which created two images, one isolating total tissue from background, and one separating elastin from tissue and background, based on the black colour of elastin after staining with Harts resorcin-fuschin. We selected a threshold to identify black-stained elastin by initially trialing the ImageJ plugin on 15 ROI from each treatment group. Once satisfied with the ability of this macro to detect elastin content separately to tissue content all ROIs were run at the set threshold in one analysis. The area of total elastin was divided by the total area of tissue to measure the proportion of elastin deposition in the lungs.

Haematoxylin and eosin (H&E)-stained sections were used for scoring epithelial sloughing (a sign of mechanical disruption of the lung epithelium^5, 6^ using a scale between 0 and 4 per ROI: no events = 0, < 5 events = 0.5, 5 - 10 events = 1 - 2, 10 - 20 events = 2.5 - 3 and > 20 events = 3.5 – 4.

Collagen was visualised with picrosirus red (PSR) stain.^7^ The birefringence of collagen was visualised using a Leica Abrio polarising microscope (512 X 512 CCD black and white camera by CRI Abrio software). The area of collagen within each ROI was normalised to area of tissue using an automated ImageJ plugin (Copyright © 2015, Keith Schulze, Monash Micro Imaging, Monash University; NIH Image).

Immunohistochemistry

Immunohistochemistry was used to identify markers outlined in Table 1. In general, sections underwent dewaxing and rehydration steps. Antigen retrieval with citrate buffer (pH 6) was used to recover antigen immunoreactivity followed by 3 x 5 minute washes with PBS. Endogenous peroxidases were blocked with 3% hydrogen peroxide (H_2_O_2_) diluted in distilled H_2_O (dH_2_O), followed by an additional 3 washes in PBS. Non-specific protein binding was blocked for 30 minutes using serum raised in the same species as the secondary antibody (e.g. normal rabbit serum (NRS) or normal goat serum (NGS)) in conjunction with bovine serum albumin (BSA). The primary antibody was immediately applied and incubated overnight at 4^o^C. Specific staining of the primary antibody was confirmed through the omission of the primary antibody.

On the second day, the primary antibody was removed with 3 x 5-minute washes with PBS. The secondary antibody was applied and slides were incubated for 60 minutes at room temperature before undergoing another 3 x 5-minute washes with PBS. Slides were incubated with an ABC kit for 30-45 minutes at room temperature and washed for 3 x 5 minutes with PBS. DAB (1 DAB tablet dissolved in 10 mL of dH_2_O and 3 µL of 30% H_2_O_2_) was applied to each section for 1–5 minutes, depending on the antibody, and stopped once brown staining was visible under a microscope. Sections were counterstained with haematoxylin, rehydrated and cover-slipped.

*Quantification of immunohistochemistry*

All sections were scanned using ImageScope (Aperio Technologies, California, USA). Sections of tissue stained for CD45, CD163, Ki67 and SP-C were analysed using ImageJ (NIH Image). Ki67 positive cells are represented as areal density of tissue by counting total cells and positive cells. The number of CD45, CD163 and SP-C cells were counted and averaged for the number of ROIs per animal. Positive stained is expressed as the average number of cells per animal.

Sections stained for α smooth muscle actin (αSMA) were analysed using ImagePro Plus (version 9.2, Build 6156, 2012-2015, Media Cybernetic, Silver Spring, MD). For each ROI the area of tissue stained for αSMA was expressed as a percentage of the total area of tissue in that ROI. All sections were analysed on sections of lung which excluded major airways or blood vessels. The % αSMA = area of total stain/by total area of tissue x 100

| **Table 1. Antibodies, dilutions indications and alterations to immunohistochemistry in paraffin-embedded lamb lung sections** | | | | |
| --- | --- | --- | --- | --- |
| **Antibody** | **Cell detected** | **Primary antibody [dilution]** | **Secondary antibody [dilution]** | **Alterations** |
| CD45 | Leukocytes | Mouse anti-CD45, Bio Rad [1:100] | Goat anti-mouse, biotinylates IgG, Vector Laboratories [1:500] | None |
| CD163 | Macrophages | Mouse anti-CD163, Acris Antibodies [1:2000] | Goat anti-mouse, biotinylates IgG, Vector Laboratories [1:200] | None |
| Ki67 | Proliferating cells in late G1-, S-, M- and G2-phases of the cell cycle | Rabbit anti-human, ThermoFisher Scientific [1:1000] | Goat anti-rabbit, biotinylates IgG, Vector Laboratories [1:200] | None |
| α smooth muscle actin | Myofibroblasts | Mouse anti-human, Dako [1:800] | Goat anti-mouse, biotinylates IgG, Vector Laboratories [1:400] | Antigen retrieval: 10mmol/L Tris, 1 mmol/L EDTA, pH9  Block: 10%BSA, 0.1%Triton  Primary: incubated in humid chamber (30min) |
| SP-C | Type II alveolar epithelial cells | Rabbit anti-SP-C [1:2000] | Goat anti-rabbit, biotinylates IgG, Vector Laboratories [1:200] | No antigen retrieval |
| IgG = immunoglobulin G; Tris = trisaminomethane; EDTA = ethylenediaminetetraacetic acid; SP = surfactant protein | | | | |

Molecular Analyses

*RNA extraction*

Snap frozen pieces of lung and liver were weighed and homogenised (Ultra Turraz T-25; Janke & Kunkel, IKA-Laboritechnik, Germany) with 1 % β-Mercaptoethanol RLT Buffer (RNeasy Kit, Qiagen, USA). Tissue lysate was centrifuged for 10 minutes at 4200 x g and the supernatant was carefully transferred into a new tube. Ethanol (70 %) was added to the supernatant and shaken vigorously and immediately applied to an RNeasy column for centrifugation for 5 minutes at 4200 x g; flow through was discarded. DNase treatment was carried out in the same column to avoid any DNA contamination in the RNA sample. RW1 buffer was applied to the column before centrifugation for 3 minutes at 4200 x g; flow through was discarded. DNase mix was applied to the column and incubated for 15 minutes at room temperature before addition of another volume of RW1 buffer and incubation for a further 1 minute at room temperature. The samples were then centrifuged for 5 minutes at 4200 x g; flow through was discarded. Two washes with RPE buffer followed and flow through was discarded. The sample was then eluted twice in RNase-free water with centrifugation for 3 minutes at 4200 x g. The flow through then contained RNA. RNA concentration was determined using spectrophotometry (NanoPhotometer® N50; Germany). Integrity of RNA was determined by gel electrophoresis and RNA was stored at -80 ºC.

For gel electrophoresis, 2 µL of RNase-free water and 2 µL of RNA sample were combined and heated at 65 ºC for 5 minutes to denature RNA. Loading buffer (2 µL; Bromophenol Blue, Xylene cyanol FF, ThermoFisher) was added to each sample before it was loaded into wells of a 1 % agarose gel (0.5 g agarose; Agarose, Scientifix, France). Gel electrophoresis was performed for 30-45 minutes at 90 V, or until bands were sufficiently separated. The gel was visualised and photographed using an ultraviolet transilluminator (Molecular Imager ChemiDox XRS System, USA). Samples were considered sufficient if they showed 28S and 18S ribosomal RNA bands and sometimes a fainter 5S band, with no smearing or other bands present.

*Complimentary DNA synthesis*

Complimentary DNA (cDNA) was transcribed from RNA using the Superscript III reverse transcription kit (Invitrogen, Australia) with provided reagents. RNA was diluted with RNase free water, 1 µL of random primers and 1 µL of dNTPs (single unites of DNA; 10 mM) and heated for 5 minutes at 65ºC to denature RNA. Reverse transcription was completed by addition of 4 µL of 5 x First-Strand buffer, 1 µL of dithiothreitol (DDT; a reducing reagent), 1 µL of RNaseOUT (recombinant RNase inhibitor) and 1 µL of Superscript III Reverse Transcriptase to each sample. The combined mixture was incubated at room temperature for 5 minutes, then at 50 ºC for 60 minutes, then was heat-activated at 70 ºC for 15 minutes and at 4 ºC until stored at -20 ºC.

*Fluidigm® Taqman® RT-PCR gene expression assay*

TaqMan probes were obtained from ThermoFisher or if unavailable were designed with help from the ThermoFisher technical team using NCBI BLAST (https://www.ncbi.nlm.nih.gov/tools/primer-blast). TaqMan probes IL-8 and SP-D were designed.

TaqMan RT-PCR assays are outlined elsewhere.^8^ Initially, a quality control (QC) report was completed in order to determine the integrity of cDNA. During the QC, samples were considered to be clean (no gDNA contamination) if Ct values were above 30. The samples then underwent a pre-amplification phase, where 3.75µL of Sample Pre Mix (Life Technologies TaqMan® PreAmp Master Mix and Pooled Taqman assays) was combined with 1.25 µL of each cDNA sample. All samples were pre-amplified over 14 cycles. Following pre-amplification, samples were diluted 1:5 by the addition of 20 µL Tris EDTA buffer pH 8.0 to the final 5 µL volume for a total volume of 25 µL.

For completion of the full Fluidigm® TaqMan® gene expression assay, each target gene was run in triplicate cDNA samples on an ABI Prism 7900HT Real-Time PCR System (PE Applied Biosystems). PCR parameters for were 50 ºC for 2 minutes, 95 ºC for 10 minutes, 40 cycles of 95 ºC for 15 seconds and 60 ºC for 1 minute. Two RT-negative samples were prepared by exclusion of Superscript III Reverse Transctiptase during the formation of cDNA. This RT-negative sample was included to test for genomic DNA contamination within the samples.

*Fluidigm® Taqman® gene expression analysis*

Ct values were generated for each cDNA sample and provided by the MHTP Medical Genomics Facility. We analysed Ct values using qBase+ software, version 3.1.^9^ We selected reference gene ribosomal (r)18S using qBase+ geNorm algorithm for assessing stability of reference genes and optimal number of reference genes.^10^ The expression of all genes are expressed as calibrated normalized relative quantity (CNRQ; i.e. normalized to r18S and expressed relative to Sal/Sal) where Sal/Sal values are normalized to ~0.00

**Statistics**

*In vitro* analyses

Apoptotic data were analysed using 2-way ANOVA with Holm-Sidak’s multiple comparison test. Wound healing assays and phagocytosis assays were compared using a one-way ANOVA with Tukey’s post hoc test. Negative control samples were not included in statistical analysis of phagocytosis data.

Physiological analyses

We identified < 5 % of physiological data points were missing and utilised sequential regression modelling to impute values (SPSS v. 24, IBM). Serial physiological data were analysed using two-way repeated measures ANCOVA (SPSS, IBM). Pearson’s correlations were used to identify covariates (SPSS, IBM). Year of delivery was used as a covariate in all physiological analyses. Additional covariates are stated where included. Cord blood gas measurements were analysed separately to serial postnatal ABGs. Post-hoc comparisons were made using the Holm-Sidak method. Histological data are expressed as mean ± SD and were analysed using Kruskal-Wallis with Dunn’s multiple comparisons post-hoc test (GraphPad Prism version 7 for Mac OS X). TaqMan® data are expressed as mean ± SD, relative to the Sal/Sal group.

**References**

1. Murphy S, Rosli S, Acharya R, Mathias L, Lim R, Wallace E, Jenkin G. Amnion epithelial cell isolation and characterization for clinical use. Curr Protoc Stem Cell Biol. 2010;Chapter 1:Unit 1E.6.

2. N.I.H. Image J: Image processing and analyses in Java. [

3. Kramer BW, Moss TJ, Willet KE, Newnham JP, Sly PD, Kallapur SG, Ikegami M, Jobe AH. Dose and time response after intraamniotic endotoxin in preterm lambs. Am J Respir Crit Care Med. 2001;164(6):982-8.

4. Willet KE, Jobe AH, Ikegami M, Newnham J, Brennan S, Sly PD. Antenatal endotoxin and glucocorticoid effects on lung morphometry in preterm lambs. Pediatr Res. 2000;48(6):782-8.

5. Hillman NH, Kallapur SG, Pillow JJ, Moss TJ, Polglase GR, Nitsos I, Jobe AH. Airway injury from initiating ventilation in preterm sheep. Pediatr Res. 2010;67(1):60-5.

6. Robertson B. Pathology of neonatal surfactant deficiency. Perspect Pediatr Pathol. 1987;11:6-46.

7. Polglase GR, Barbuto J, Allison BJ, Yawno T, Sutherland AE, Malhotra A, Schulze KE, Wallace EM, Jenkin G, Ricardo SD, Miller SL. Effects of antenatal melatonin therapy on lung structure in growth-restricted newborn lambs. J Appl Physiol (1985). 2017;123(5):1195-203.

8. Medhurst AD, Harrison DC, Read SJ, Campbell CA, Robbins MJ, Pangalos MN. The use of TaqMan RT-PCR assays for semiquantitative analysis of gene expression in CNS tissues and disease models. J Neurosci Methods. 2000;98(1):9-20.

9. Hellemans J, Mortier G, De Paepe A, Speleman F, Vandesompele J. qBase relative quantification framework and software for management and automated analysis of real-time quantitative PCR data. Genome Biol. 2007;8(2):R19.

10. Vandesompele J, De Preter K, Pattyn F, Poppe B, Van Roy N, De Paepe A, Speleman F. Accurate normalization of real-time quantitative RT-PCR data by geometric averaging of multiple internal control genes. Genome Biol. 2002;3(7):RESEARCH0034.
