# Supplementary figures and images for: The effect of human amnion epithelial cells on lung development and inflammation in preterm lambs exposed to antenatal inflammation
Source: PLoS One. 2021 Jun 25;16(6):e0253456. doi: 10.1371/journal.pone.0253456 (PMC8232434; doi:10.1371/journal.pone.0253456)

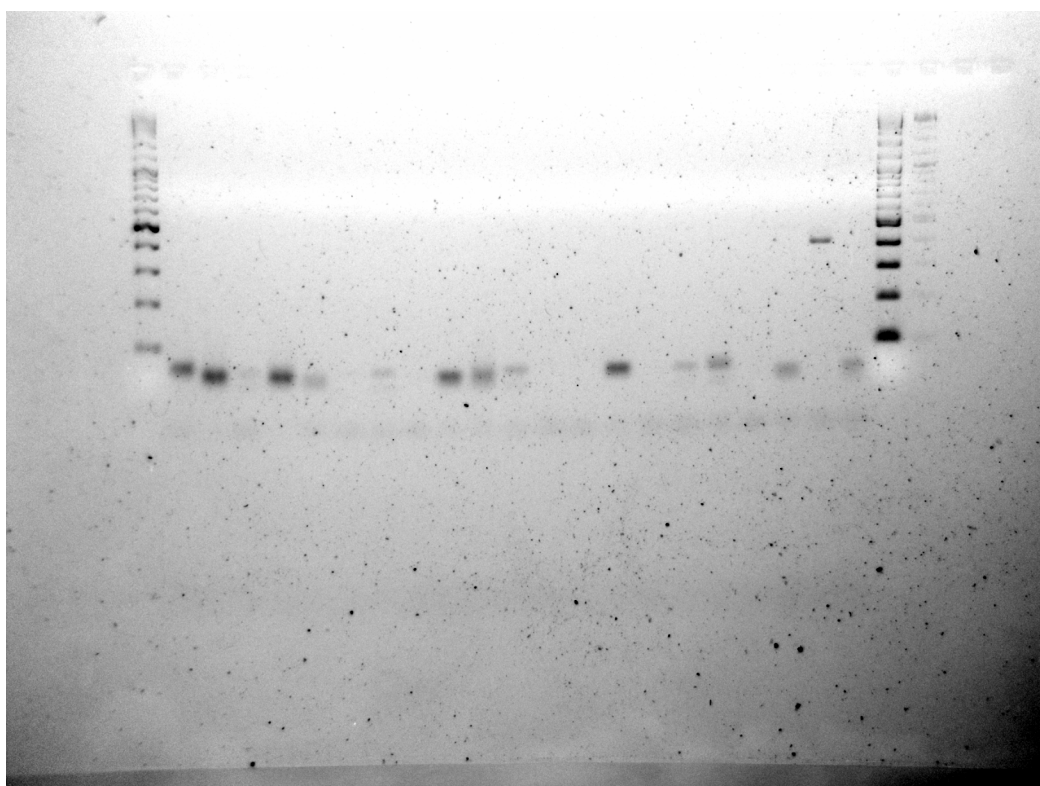

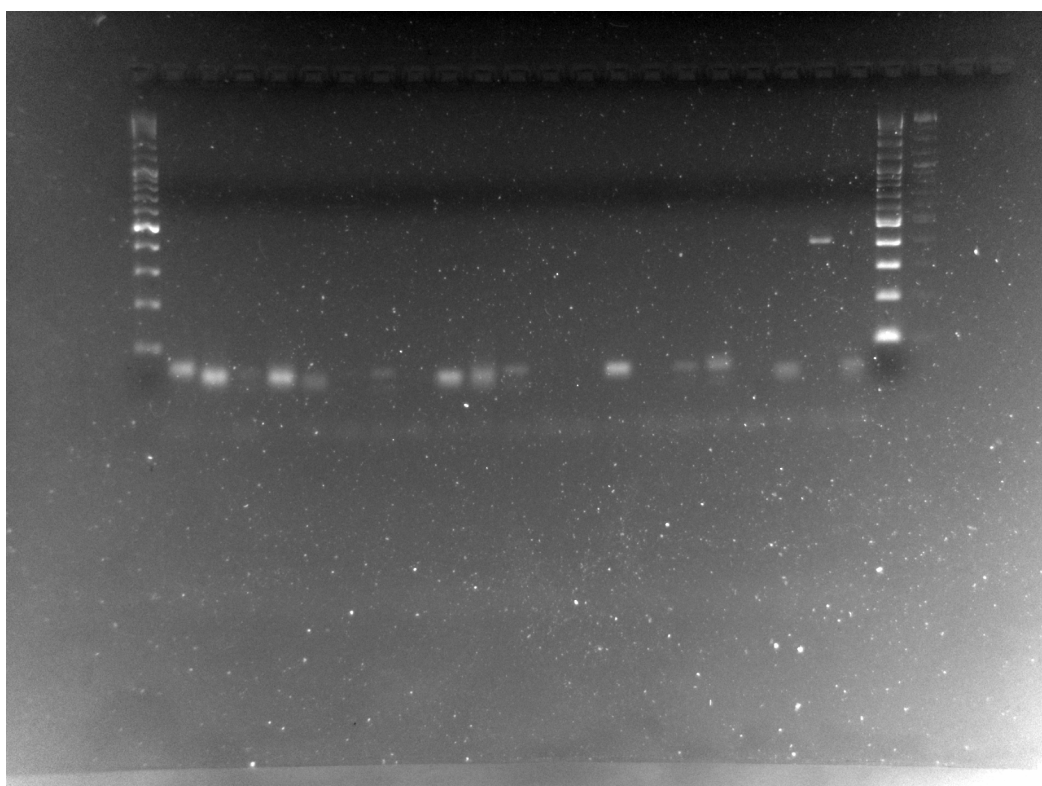

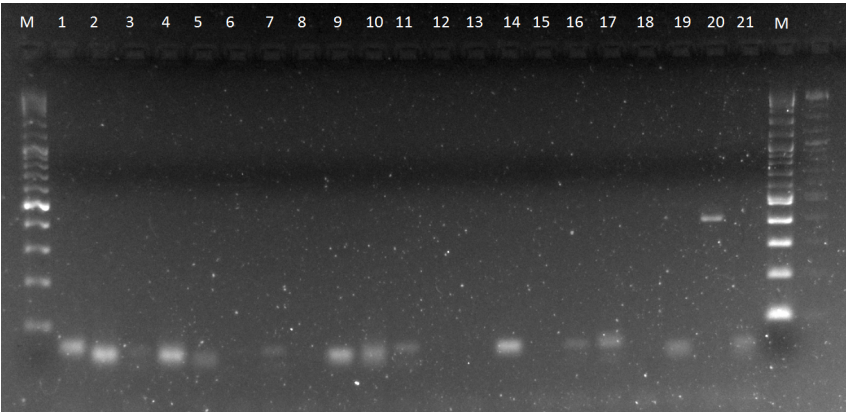

Supplement: S1 Raw image — Lane 20 is positive control. Lane 21 is a negative control. (PDF) [file pone.0253456.s008.pdf]
