# Supplementary material for: The effect of human amnion epithelial cells on lung development and inflammation in preterm lambs exposed to antenatal inflammation
Source: PLoS One. 2021 Jun 25;16(6):e0253456. doi: 10.1371/journal.pone.0253456 (PMC8232434; doi:10.1371/journal.pone.0253456)
Supplement: Attachment — Submitted filename: Response to reviewers.docx [file pone.0253456.s009.docx]

Dear Editor,

Thank you for your consideration of our manuscript “The effect of human amnion epithelial cells on lung development and inflammation in preterm lambs exposed to antenatal inflammation”. We believe we have adjusted this manuscript to meet the journal requirements and comments from the editor and reviewers 1 and 2. Please note that our original blot image is attached as a separate PDF file “S4_raw_image” and as part of supplementary figures (S4 Fig.).

Please find our responses to individual reviewers below. We have attached a marked-up version of the manuscript with changes in red text. We have addressed concerns of funding bodies raised by the editor in text on p. 33, line starting 702.

Additionally, please note that we inadvertently omitted one author, Dr. Graeme Polglase. We recognise the addition of this author and this author has reviewed the manuscript. Polglase’s contributions to the manuscript are outlined in red on p.ii, line 27-8. All authors support the alterations and submission of this manuscript.

We greatly appreciate your consideration of this manuscript.

Kind Regards,

Paris Papagianis and co-authors.

**Reviewer #1:**

The authors hypothesized that human amnion epithelial cells (hAECs) would reduce lung injury in preterm lambs-exposed to antenatal inflammation and investigated the effects of a single dose of intravenous hAECs on lung development and respiratory requirement. The authors found that hAECs administration at birth did not reduce respiratory requirements or lung development in preterm lambs exposed to antenatal inflammation. This study has many data and the results do not confirm the hypothesis and the connection between in vitro and animal study was not evident.

General concerns:
1. This study presents many data and most data did not show clear evidence of positive effects.

We believe that the data presented provide important insight into the use of translational studies investigating cellular therapies for lung disease. We use a clinically relevant preterm intensive care model which recapitulates human practice to demonstrate that while the impact of hAECs on ventilator requirements is minimal the positive effects on lung parenchyma are apparent. Additionally, we analyse the impact of contemporary NICU care in the context of chorioamnionitis (a common precursor to preterm birth and NICU). The outcomes of these studies are informing the planning and design of clinical trials using hAECs in human infants.

2. The conclusions in Abstract was not supported by the results: Postnatal administration of a single dose of hAECs activates the pulmonary immune system without changing ventilator requirements in preterm lambs born after intrauterine inflammation. However, activation of pulmonary immune system was not described in the manuscript.

We describe markers of pulmonary inflammation in Fig. 5. We have removed the word “activation” from the abstract and replaced with “stimulates a pulmonary immune response”.
3. Methods: Please describe how the cell numbers of hAECs (30 × 106) was decided in this study.

We chose a hAEC dose of roughly 10 million cells per kilo, based on the average weight of preterm lambs at 128 dGA (~3kg). This dosing approach for hAECs was in line with a planned Phase 2 clinical trial for hAECs in preterm infants with BPD when our study was conducted.

4. Please describe and discuss the rationale to measure hepatic gene expression in lung injury model.

Although the focus of this study was on ventilator requirements and lung development/maturation, we opportunistically looked at markers for systemic inflammation. This analysis of hepatic gene expression as an indicator of systemic inflammation informed our conclusion that hAECs were administered safely in our preterm lambs.

5. Methods: The animal numbers were not consistent. (LPS/Sal, n=10; or Sal/Sal, n=9) in Abstract. Preterm lamb studies on page 7, Ultrasound-guided intra-amniotic (IA) injection of lipopolysaccharide (LPS;4 mg; 2 mg/mL; Escherichia coli 055:B5; Sigma-Aldrich, NSW, Australia; n=10) or saline (n=10) was performed at 126 days’ GA.

Thank you. Unfortunately, this is typological error in the abstract. Saline/Saline = 9. This group size number has been amended for the Saline/Saline group throughout the manuscript.
6. Methods: The methods to collect the Physiology variables in Table 2 were not described.

All the methodology used to collect physiological variables are described in detail in the supplementary methods due to word restrictions in the main paper. A detailed methodology for data collection of physiological variables is cited in the general postnatal management section of our methodology in the main manuscript (line 152 onwards).
7. Results: Please insert (Table 1) in the third paragraph on page 15.

Please find Table 1 at line 242

8. Figure Legend 1: “^Signifies P<0.05 between Sal/Sal and LPS/hAECs” was not shown in the Figure 1. Please describe the meaning of **.

Figure legends are now adjusted to define all symbols in each figure. ^Signifies P<0.05 between Sal/Sal and LPS/Sal. *Signifies P<0.05 between Sal/Sal and LPS/hAECs ^&^Time signifies P<0.05 change over 1-6 days, not between treatment groups.” There is no use of ** in any of the figures, and these symbols are consistent for all subsequent figures. Additionally, we have simplified the description of the colour coding in figure 2 and enhanced the labelling in Figures 1 and 5 to improve readability.

**Reviewer #2:**

The study highlights the role of inflammatory axis in the genesis of CLD. By and large the manuscript reads well. I would like to add couple of comments for minor revision

1) Explicitly state the three groups based on the antenatal- post natal interventions - e.g. group A (LPS/S); Group B (S/S) to improve clarity

The use of A, B and C for treatment groups may be more confusing as the reader has to remember what each represents. We abbreviate Saline to Sal and human amnion epithelial cells to hAEC, as is standard within the literature.

2) The abstract, discussion, conclusions- explicitly state the effect of hAEC on inflammatory cytokines- while it states hAEC influence pulmonary immunology -- for better clarity state the effect of hAEC on pro and anti-inflammatory cytokine response. This aspect is also discussed appropriately in the limitations

We have adjusted the abstract as per reviewer #1 comment which we believe also meets the requirements of this comment. The discussion and conclusion have also been changed accordingly. The discussion:

- Line 398 “level of pro- or anti- inflammatory cytokine gene expression in the lungs”
- Line 463: “increases the expression of the anti-inflammatory cytokine, IL-10”
- Line 471-3: “However, we did not see reduced pro-inflammatory cytokine expression in preterm lambs receiving hAEC after antenatal LPS exposure in this study, despite increased IL-10 compared to LPS/Sal lambs.”

The conclusion has been altered: “level of pro- or anti- inflammatory cytokine gene expression in the lungs”
